# Alterations in fecal short chain fatty acids (SCFAs) and branched short-chain fatty acids (BCFAs) in men with benign prostatic hyperplasia (BPH) and metabolic syndrome (MetS)

**DOI:** 10.18632/aging.202968

**Published:** 2021-04-13

**Authors:** Weronika Ratajczak, Arnold Mizerski, Aleksandra Rył, Marcin Słojewski, Olimpia Sipak, Małgorzata Piasecka, Maria Laszczyńska

**Affiliations:** 1 Department of Histology and Development Biology, Pomeranian Medical University in Szczecin, Szczecin 71-210, Poland; 2 Department of General Pharmacology and Pharmacoeconomics, Pomeranian Medical University in Szczecin, Szczecin 71-210, Poland; 3 Department of General and Gastroentereological Surgery, Pomeranian Medical University in Szczecin, Szczecin 71-252, Poland; 4 Department of Medical Rehabilitation and Clinical Physiotherapy, Pomeranian Medical University in Szczecin, Szczecin 71-210, Poland; 5 Department of Urology and Urological Oncology, Pomeranian Medical University in Szczecin, Szczecin 70-111, Poland; 6 Department of Obstetrics and Pathology of Pregnancy, Pomeranian Medical University in Szczecin, Szczecin 71-210, Poland

**Keywords:** short-chain fatty acids, benign prostatic hyperplasia (BPH), metabolic syndrome (MetS), gut microbiota, microbiota-gut-prostate axis

## Abstract

Gut microbiome-derived short-chain fatty acids (SCFAs) emerge in the process of fermentation of polysaccharides that resist digestion (dietary fiber, resistant starch). SCFAs have a very high immunomodulatory potential and ensure local homeostasis of the intestinal epithelium, which helps maintain the intestinal barrier. We analyzed the association between stool SCFAs levels acetic acid (C 2:0), propionic acid (C 3:0), isobutyric acid (C 4:0i), butyric acid (C 4:0n), isovaleric acid (C 5:0i) valeric acid (C 5:0n), isocaproic acid (C 6:0i), and caproic acid (C 6:0n)) in aging man with benign prostatic hyperplasia (BPH) and healthy controls. The study involved 183 men (with BPH, n = 103; healthy controls, n = 80). We assessed the content of SCFAs in the stool samples of the study participants using gas chromatography. The levels of branched SCFAs (branched-chain fatty acids, BCFAs): isobutyric acid (C4:0i) (p = 0.008) and isovaleric acid (C5:0i) (p < 0.001) were significantly higher in patients with BPH than in the control group. In healthy participants isocaproic acid (C6:0i) predominated (p = 0.038). We also analyzed the relationship between stool SCFA levels and serum diagnostic parameters for MetS. We noticed a relationship between C3:0 and serum lipid parameters (mainly triglycerides) in both healthy individuals and patients with BPH with regard to MetS. Moreover we noticed relationship between C4:0i, C5:0i and C6:0i and MetS in both groups. Our research results suggest that metabolites of the intestinal microflora (SCFAs) may indicate the proper function of the intestines in aging men, and increased BCFAs levels are associated with the presence of BPH.

## INTRODUCTION

Benign prostatic hyperplasia (BPH) is one of the most commonly diagnosed urological diseases in men over 50 years of age. BPH is characterized by prostatic stromal cell proliferation, leading to prostatic bladder obstruction (BOO) and lower urinary tract symptoms (LUTS), which together reduce quality of life (QoL). The development of BPH is very often associated with the existence of comorbidities, such as diabetes, cardiovascular diseases, and even neurological diseases [1]. Many studies also indicate a relationship between the metabolic syndrome (MetS) and the risk of LUTS and BPH [2–4]. MetS is defined as the combination of obesity, dyslipidemia, hyperglycemia, high blood pressure and insulin resistance. The factor that contributes to the initiation of pathological changes in the prostate, and consequently its benign hyperplasia, is chronic inflammation resulting, among others, from metabolic disorders [5]. Moreover, MetS is accompanied by sex hormone disorders, which is also one of the etiological factors of BPH [6].

The intestinal microflora is one of the elements of the bacterial ecosystem in mammals. The microorganisms that inhabit the gut are one of the key elements involved in modulating the immune response from the moment of birth. More and more research is currently being carried out on the effect of bacterial metabolites, including short-chain fatty acids (SCFA), on homeostasis, not only in the intestinal microenvironment, but also in cells and tissues of other organs. SCFAs are a group of compounds made up of six carbon atoms (C1 - C6), with the majority of acids being: butyric acid (C4), propionic acid (C3), and acetic acid (C2). These acids are present in humans in certain amounts, but their proportions may change depending on the diet, medications, age, and diseases. The concentrations of SCFAs depend on the ratio of bacteria inhabiting the intestines, and disorders of the intestinal microflora (dysbiosis), which affects the amount of SCFAs produced. These fatty acids play a significant role in maintaining homeostasis—they ensure the appropriate pH of the intestinal microenvironment [7], participate in the maintenance of the intestinal barrier, and are a source of energy for the intestinal epithelium and hepatocytes [8]. These acids are also inhibitors of histone deacetylases (HDACs), thanks to which they regulate epigenetic processes [9] and have an immunomodulating effect not only locally but also in distant tissues of the body [10, 11].

The method of assessing SCFAs in patient stool samples is a quick, cheap and common technique that allows the determination of intestinal microflora disorders [12–14]. Although this method does not assess the composition of the microbiota, it determines the content of its metabolite products, which also allows for an indirect assessment of the intestinal microbiota [15]. In addition, information is also obtained about the type of fermentation taking place in the gut that results in the production of SCFAs [16]. SCFA levels vary depending on the composition of the intestinal microflora and food intake [12]. Differences in the analytical methods and methodology of preparing material for research cause difficulties interpreting the results obtained.

So far, disturbances in the intestinal microflora and its impact on inflammation and prostate diseases have not yet been thoroughly analyzed. The influence of SCFAs on the development of BPH has also not been studied. Only a few publications on the impact of the intestinal microflora on the prostate can be found in the literature. They mainly concern the influence of intestinal bacteria on the synthesis of metabolites and androgens, which may be associated with the development of prostate cancer in humans [17, 18]. There are also reports of the impact of inflammatory bowel disease (IBD) on the risk of prostate cancer [19]. So far, differences in the composition of the intestinal microflora have only been confirmed in a pilot study, in which the composition of the intestinal microflora was analyzed in patients with prostate cancer, and with BPH [20]. The results of the research by Liss et al. [17] indicate that bacteria predominating in men diagnosed with prostate cancer (PCa) were *Bacteroides* and *Streptococcus spp*. As reviewed by Shah [21] BPH is not a precursor stage to prostate cancer. Moreover, these two diseases affect different areas of the gland. Nevertheless, it turns out that it is possible to identify risk factors that are common to the development of BPH and PCa [22]. They include genetic factors, androgen signalling, oxidative stress, [21] and inflammation [23, 24]. It is indicated that chronic prostate inflammation predisposes to BPH and PCa, and is not always caused by a bacterial infection, but can be associated with low-grade systemic inflammation [25]. The exact mechanism by which the intestinal microflora affects the prostate gland has not so far been fully elucidated. It seems very likely, however, that disturbed intestinal microflora does not directly affect the prostate gland, but contributes to the development of chronic systemic inflammation. Inflammatory cells and factors (including cytokines) from the intestinal environment, along with the circulation, can get into the gland and there cause ‘local’ inflammation and stimulate the growth factors of the prostate stroma, which in turn may lead to prostatic hyperplasia. The influence of the intestinal microflora and its metabolites entering the systemic circulation has been confirmed in studies on the urinary [26], nervous [27], and respiratory [28] systems, as well as autoimmune diseases [29].

The main aim of this study was to compare the profile of SCFAs between healthy individuals and patients diagnosed with BPH, with regard to MetS as a factor predisposing to the development of prostate hyperplasia. Our study is the first to show changes in the tested SCFAs levels between these two groups. Nevertheless, further research is needed (including testing in animal models) to determine whether there is a ‘microbiota-gut-prostate axis’ and whether the intestinal microflora and its metabolites contribute to the development of BPH.

## RESULTS

### Factors proving the presence of BPH

The study involved 183 men. The control group included 80 healthy men without BPH (mean PV = 22 ml ± 6.9, mean Q_max_ = 20.08 ml/s ± 9). The study group consisted of 103 patients with a diagnosis of BPH, qualified for transurethral resection of the prostate (TURP) (mean PV = 61.95 ml ± 29, mean Q_max_ = 10.39 ml/s ± 6.66).

There were statistically significant differences between the groups (Table 1) regarding the prostate volume (p < 0.001), Q_max_ (p < 0.001), the results of the IPSS questionnaire (p < 0.001), QoL (quality of life score) (p < 0.001), the results of the ADAM (Androgen Deficiency in Aging Men) questionnaire (p < 0.001).

**Table 1:** Characteristics of the control group (healthy volunteers, without BPH) and the study group (patients diagnosed with BPH).

**Parametr**	**Healthy volunteers (without BPH) (n=80)**						**Patients with BPH (n=103)**					**p-value**
	**Mean**	**Median**	**Min**	**Max**	**SD**		**Mean**	**Median**	**Min**	**Max**	**SD**	
**Age** [years]	54.66	54.00	45.00	72.00	6.52		66.46	67.00	49.00	79.00	6.50	< 0.001*
**PV** [ml]	22.15	21.90	11.60	33.00	5.34		65.20	60.00	35.00	120.00	20.50	< 0.001*
**Q_max_** [ml/s]	20.08	19.20	6.30	42.10	9.01		10.39	9.10	2.00	40.00	6.66	< 0.001*
**IPSS**	3.31	3.00	0.00	7.00	1.84		19.76	21.00	3.00	35.00	7.79	< 0.001*
**QoL**	1.43	1.00	0.00	4.00	1.07		3.30	3.50	0.00	5.00	1.25	< 0.001*
**ADAM**	3.61	3.00	0.00	8.00	2.24		0.84	1.00	0.00	1.00	0.37	< 0.001*
**TG** [mmol/l]	163.07	156.80	110.49	306.67	40.37		176.32	157.58	114.05	815.43	84.51	0.250
**Cholesterol** [mg/dl]	215.13	208.39	136.29	320.28	39.31		204.53	200.75	143.49	404.10	38.30	0.035*
**HDL** [mg/dl]	44.76	43.98	30.32	76.62	10.64		55.76	51.35	41.70	383.49	34.00	< 0.001*
**LDL** [mg/dl]	137.76	133.17	56.32	232.63	40.48		117.24	110.22	1.79	321.07	39.69	0.001*
**FPG** [mg/dl]	100.65	99.42	85.43	161.13	10.51		79.14	80.19	49.43	109.17	13.67	< 0.001*

BPH – benign prostatic hyperplasia; n – number; Min – minimum; Max – maximum; SD – standard deviation; PV – prostate volume, Q_max_ - maximum flow rate in uroflowmetry; IPSS - International Prostate Symptom Score; QoL - Quality of Life score; ADAM -Androgen Deficiency in Aging Men questionnaire; TG – triglycerides; HDL – high density lipoprotein; LDL – low density lipoprotein; FPG – fasting plasma glucose; p – statistical significance; * - statistical significant parameter.

Anthropometric measurements were also performed in the patients. Healthy patients had higher body weight and height than patients with BPH (88.854 vs. 84.825, p = 0.038; 1.767 vs. 1.742, p = 0.005). There were no statistically significant differences in patients' BMI (28.480 vs. 28.051, p = 0.498).

### Biochemical parameters

Serum biochemical parameters in the control and the study groups were also measured (Table 1). The levels of total cholesterol (p = 0.035), LDL cholesterol (p = 0.001) and glucose (p < 0.001) were statistically significantly higher in healthy patients. At the same time, statistically significantly lower HDL cholesterol levels (p < 0.001) were found in patients without BPH. There was no relationship in TG levels between the groups, although their mean values were higher in patients with BPH.

### SCFA levels in the study and the control groups with regard to MetS

The study shows that the control group had higher levels of acetic and propionic acids, however these results were not statistically significant. Whereas, the levels of isocaproic acid were statistically significantly higher (p = 0.038). Patients with BPH, on the other hand, had statistically significant higher levels of branched-chain SCFAs (BCFAs): isobutyric acid (p = 0.001) and isovaleric acid (p < 0.001) (Table 2).

**Table 2:** Short-chain fatty acids in patients in the control group (healthy volunteers, without BPH) and the study group (patients diagnosed with BPH), and depending on the presence of MetS.

**SCFAs (%)**	**Healthy volunteers (without BPH) (n=80)**						**Patients with BPH (n=103)**					**p-value**
	**Mean**	**Median**	**Min**	**Max**	**SD**		**Mean**	**Median**	**Min**	**Max**	**SD**	
**C 2:0**	35.148	35.210	17.408	57.556	6.792		33.317	32.898	15.205	61.909	7.680	0.059
**C 3:0**	21.567	20.809	8.710	47.941	6.805		20.531	19.662	0.903	36.581	6.298	0.330
**C 4:0 i**	3.814	3.726	0.266	8.293	1.947		4.695	4.702	0.370	16.163	2.226	0.008*
**C 4:0 n**	24.006	22.394	3.804	47.489	8.125		23.216	23.734	6.094	47.467	8.395	0.631
**C 5:0 i**	6.911	6.730	0.216	19.616	4.201		9.499	9.221	0.450	33.867	4.990	< 0.001*
**C 5:0 n**	5.729	5.637	0.194	11.426	2.189		6.119	6.372	0.655	16.167	2.645	0.166
**C 6:0 i**	0.359	0.174	0.015	10.799	1.198		0.186	0.142	0.023	0.741	0.149	0.038*
**C 6:0 n**	2.057	1.618	0.047	7.690	1.986		2.120	1.396	0.088	10.709	2.132	0.732
**SCFAs (%)**	**Healthy volunteers (without BPH) with MetS (n=36)**						**Patients with BPH with Mets (n=42)**					**p-value**
	**Mean**	**Median**	**Min**	**Max**	**SD**		**Mean**	**Median**	**Min**	**Max**	**SD**	
**C 2:0**	33.535	34.113	17.408	43.210	5.905		33.373	32.372	19.923	61.909	8.556	0.524
**C 3:0**	24.385	23.201	10.807	47.941	7.482		21.175	20.295	4.921	36.581	7.000	0.059
**C 4:0 i**	3.573	3.199	0.266	8.293	1.767		4.489	4.409	0.438	9.394	2.154	0.044*
**C 4:0 n**	22.897	21.641	9.093	37.315	6.253		23.880	24.247	6.596	47.467	8.945	0.426
**C 5:0 i**	6.430	5.698	0.216	19.616	3.871		8.902	8.637	0.450	19.674	5.050	0.029*
**C 5:0 n**	6.333	6.264	0.685	11.426	2.392		5.826	6.287	0.795	12.820	2.611	0.480
**C 6:0 i**	0.492	0.154	0.018	10.799	1.773		0.141	0.104	0.028	0.404	0.094	0.019*
**C 6:0 n**	1.955	1.075	0.047	7.690	2.146		1.943	0.881	0.123	5.911	1.955	0.869

BPH – benign prostatic hyperplasia; MetS – metabolic syndrome; n – number; Min – minimum; Max – maximum; SD – standard deviation; SCFAs – short-chain fatty acids; C2:0 - acetic acid; C3:0 - propionic acid; C4:0i - isobutyric acid; C4:0n - butyric acid; C5:0i - isovaleric acid; C5:0n - valeric acid; C6:0i - isocaproic acid; C 6:0n - caproic acid; p – statistical significance; * - statistical significant parameter.

Additionally, patients with BPH and MetS had significantly higher stool BCFAs levels—isobutyric acid (p = 0.044) and isovaleric acid (p = 0.029)—compared to healthy controls. Healthy patients with MetS had significantly higher levels of isocapronic acid (p = 0.019) in comparison to patients with BPH and MetS (Table 2).

Healthy controls without MetS had significantly lower stool levels of propionic acid (C3:0) (p = 0.002) and valeric acid (C5:0n) (p = 0.025) in comparison to healthy controls with MetS (Table 3). Patients diagnosed with BPH without MetS had significantly higher stool levels of isocapronic acid (p = 0.034) (Table 3).

**Table 3:** Short-chain fatty acids in control patients and in patients diagnosed with BPH, depending on the presence of MetS.

**SCFAs (%)**	**Healthy volunteers (without BPH) (n=80)**											**p-value**
	**Without MetS (n=44)**						**With Mets (n=36)**					
	**Mean**	**Median**	**Min**	**Max**	**SD**		**Mean**	**Median**	**Min**	**Max**	**SD**	
**C 2:0**	36.468	35.730	22.598	57.556	7.240		33.535	34.113	17.408	43.210	5.905	0.116
**C 3:0**	19.262	19.892	8.710	32.297	5.238		24.385	23.201	10.807	47.941	7.482	0.002*
**C 4:0 i**	4.010	4.085	0.438	7.801	2.082		3.573	3.199	0.266	8.293	1.767	0.294
**C 4:0 n**	24.914	24.461	3.804	47.489	9.359		22.897	21.641	9.093	37.315	6.253	0.431
**C 5:0 i**	7.304	7.290	0.451	17.440	4.458		6.430	5.698	0.216	19.616	3.871	0.331
**C 5:0 n**	5.235	5.482	0.193	9.567	1.895		6.333	6.264	0.685	11.426	2.392	0.025*
**C 6:0 i**	0.251	0.174	0.015	1.025	0.234		0.492	0.154	0.018	10.799	1.773	0.525
**C 6:0 n**	2.141	2.274	0.112	7.475	1.865		1.955	1.075	0.047	7.690	2.146	0.557
**SCFAs (%)**	**Patients with BPH (n=103)**											**p-value**
	**Without MetS (n=61)**						**With MetS (n=42)**					
	**Mean**	**Median**	**Min**	**Max**	**SD**		**Mean**	**Median**	**Min**	**Max**	**SD**	
**C 2:0**	33.279	33.227	15.205	49.396	7.088		33.373	32.402	19.923	61.91	8.556	0.660
**C 3:0**	20.088	19.412	0.903	35.717	5.784		21.175	20.031	4.921	36.58	7.000	0.562
**C 4:0 i**	4.838	4.723	0.370	16.163	2.281		4.489	4.413	0.438	9.39	2.154	0.492
**C 4:0 n**	22.759	23.054	6.094	39.532	8.039		23.880	24.232	6.596	47.47	8.945	0.457
**C 5:0 i**	9.911	9.554	1.944	33.867	4.948		8.902	8.842	0.450	19.67	5.050	0.336
**C 5:0 n**	6.321	6.443	0.655	16.167	2.670		5.826	6.312	0.795	12.82	2.611	0.403
**C 6:0 i**	0.217	0.167	0.023	0.7441	0.172		0.141	0.103	0.028	0.40	0.094	0.034*
**C 6:0 n**	2.242	1.578	0.088	10.709	7.253		1.943	0.822	0.123	5.911	1.955	0.388

BPH – benign prostatic hyperplasia; MetS – metabolic syndrome; n – number; Min – minimum; Max – maximum; SD – standard deviation; SCFAs – short-chain fatty acids; C2:0 - acetic acid; C3:0 - propionic acid; C4:0i - isobutyric acid; C4:0n - butyric acid; C5:0i - isovaleric acid; C5:0n - valeric acid; C6:0i - isocaproic acid; C 6:0n - caproic acid; p – statistical significance; * - statistical significant parameter.

### SCFA levels and biochemical parameters

In all patients from the control group a positive correlation was found only between propionic acid and the levels of triglycerides (R = 0.385, p < 0.05), total cholesterol (R = 0.290, p = 0.010), and LDL cholesterol (R = 0.244, p = 0.030) (Table 4). In the group of patients with BPH, a positive correlation was also demonstrated between the levels of propionic acid and triglycerides (R = 0.232, p = 0.021), and a negative correlation was observed between the levels of isocaproic acid and glucose (R = -0.272, p = 0.007) (Table 4).

**Table 4:** Correlations between the analyzed SCFAs and biochemical parameters in healthy patients from the control and study group.

**Biochemical parameters**	**Healthy volunteers (without BPH) (n= 80)** **%SCFAs**															
	**C2:0**		**C 3:0**		**C 4:0 i**		**C 4:0 n**		**C 5:0 i**		**C 5:0 n**		**C 6:0 i**		**C 6:0 n**	
	**R**	**p**	**R**	**p**	**R**	**p**	**R**	**p**	**R**	**p**	**R**	**p**	**R**	**p**	**R**	**p**
**TG** [mmol/l]	-0.068	0.551	**0.385**	< 0.05*	-0.132	0.245	-0.125	0.272	-0.115	0.312	0.152	0.181	-0.009	0.941	-0.080	0.482
**Cholesterol** [mg/dl]	-0.146	0.201	**0.290**	0.010*	-0.129	0.258	-0.037	0.746	-0.151	0.185	0.091	0.428	-0.166	0.144	-0.069	0.548
**HDL** [mg/dl]	0.200	0.077	-0.128	0.260	-0.056	0.624	-0.076	0.507	-0.072	0.527	-0.040	0.724	-0.008	0.946	0.021	0.857
**LDL** [mg/dl]	-0.198	0.080	**0.244**	0.030*	-0.073	0.520	0.003	0.980	-0.093	0.416	0.103	0.365	-0.129	0.259	-0.047	0.680
**FPG** [mg/dl]	-0.149	0.187	0.212	0.059	-0.160	0.156	0.002	0.983	-0.161	0.153	0.161	0.154	-0.037	0.745	0.103	0.361
**Biochemical parameters**	**Patients with BPH (n= 103)** **%SCFAs**															
	**C2:0**		**C2:0**		**C2:0**		**C2:0**		**C2:0**		**C2:0**		**C2:0**		**C2:0**	
	**R**	**p**	**R**	**p**	**R**	**p**	**R**	**p**	**R**	**p**	**R**	**p**	**R**	**p**	**R**	**p**
**TG** [mmol/l]	0.021	0.834	**0.232**	0.021*	-0.078	0.441	-0.028	0.782	-0.122	0.230	-0.117	0.248	0.094	0.353	-0.121	0.231
**Cholesterol** [mg/dl]	-0.035	0.729	0.040	0.692	-0.109	0.283	0.109	0.281	-0.125	0.216	-0.138	0.173	-0.106	0.296	-0.095	0.348
**HDL** [mg/dl]	-0.020	0.843	-0.058	0.571	-0.032	0.757	0.073	0.470	-0.013	0.900	0.023	0.819	-0.107	0.291	-0.019	0.855
**LDL** [mg/dl]	-0.034	0.742	-0.043	0.675	-0.095	0.351	0.107	0.294	-0.104	0.308	-0.092	0.368	-0.170	0.094	-0.049	0.635
**FPG** [mg/dl]	-0.053	0.602	0.062	0.543	-0.067	0.509	0.043	0.674	-0.079	0.436	0.021	0.836	**-0.272**	0.007*	0.026	0.802

BPH – benign prostatic hyperplasia; n – number; SCFAs – short-chain fatty acids; C2:0 - acetic acid; C3:0 - propionic acid; C4:0i - isobutyric acid; C4:0n - butyric acid; C5:0i - isovaleric acid; C5:0n - valeric acid; C6:0i - isocaproic acid; C 6:0n - caproic acid; TG – triglycerides; HDL – high density lipoprotein; LDL – low density lipoprotein; FPG – fasting plasma glucose; R - correlation coefficient; p – statistical significance; * - statistical significant parameter.

In the group of healthy controls analyzed with regard to MetS, a positive correlation between the levels of propionic acid, triglycerides (R = 0.331, p = 0.049) and total cholesterol (R = 0.399, p = 0.016) was only observed in patients diagnosed with MetS (Table 5).

**Table 5:** Correlations between the analyzed SCFAs and the biochemical parameters in healthy control patients without and with metabolic syndrome.

**Biochemical parameters**	**Healthy volunteers (without BPH) without MetS (n=44)** **%SCFAs**															
	**C2:0**		**C 3:0**		**C 4:0 i**		**C 4:0 n**		**C 5:0 i**		**C 5:0 n**		**C 6:0 i**		**C 6:0 n**	
	**R**	**p**	**R**	**p**	**R**	**p**	**R**	**p**	**R**	**p**	**R**	**p**	**R**	**p**	**R**	**p**
**TG** [mmol/l]	0.041	0.794	0.224	0.149	-0.010	0.949	-0.071	0.652	0.028	0.860	-0.063	0.686	0.137	0.383	-0.067	0.671
**Cholesterol** [mg/dl]	0.067	0.669	-0.078	0.620	0.018	0.907	-0.008	0.957	-0.023	0.883	0.053	0.735	-0.033	0.831	0.088	0.573
**HDL** [mg/dl]	0.296	0.054	-0.078	0.617	-0.151	0.333	-0.145	0.353	-0.154	0.323	-0.026	0.870	-0.183	0.239	0.197	0.205
**LDL** [mg/dl]	-0.090	0.564	-0.039	0.806	0.113	0.469	0.031	0.845	0.076	0.627	0.090	0.566	0.080	0.609	0.005	0.974
**FPG** [mg/dl]	-0.204	0.185	0.151	0.329	-0.150	0.332	0.123	0.425	-0.175	0.256	0.013	0.931	0.004	0.979	0.115	0.458
**Biochemical parameters**	**Healthy volunteers (without BPH) with MetS (n=36)** **%SCFA**															
	**C2:0**		**C 3:0**		**C 4:0 i**		**C 4:0 n**		**C 5:0 i**		**C 5:0 n**		**C 6:0 i**		**C 6:0 n**	
	**R**	**p**	**R**	**p**	**R**	**p**	**R**	**p**	**R**	**p**	**R**	**p**	**R**	**p**	**R**	**p**
**TG** [mmol/l]	-0.013	0.939	**0.331**	0.049*	-0.171	0.318	-0.128	0.455	-0.172	0.316	0.104	0.546	0.069	0.688	-0.134	0.437
**Cholesterol** [mg/dl]	-0.240	0.158	**0.399**	0.016*	-0.233	0.171	0.033	0.847	-0.245	0.150	0.009	0.957	-0.187	0.274	-0.076	0.659
**HDL** [mg/dl]	0.011	0.947	0.019	0.912	-0.033	0.846	0.021	0.904	-0.077	0.654	0.053	0.758	0.132	0.444	-0.183	0.284
**LDL** [mg/dl]	-0.243	0.154	0.323	0.055	-0.178	0.299	0.046	0.791	-0.188	0.271	0.044	0.801	-0.220	0.197	0.020	0.909
**FPG** [mg/dl]	0.118	0.492	0.023	0.896	-0.107	0.535	-0.107	0.533	-0.081	0.637	0.146	0.397	-0.009	0.960	0.212	0.214

BPH – benign prostatic hyperplasia; MetS – metabolic syndrome; n – number; SCFAs – short-chain fatty acids; C2:0 - acetic acid; C3:0 - propionic acid; C4:0i - isobutyric acid; C4:0n - butyric acid; C5:0i - isovaleric acid; C5:0n - valeric acid; C6:0i - isocaproic acid; C 6:0n - caproic acid; TG – triglycerides; HDL – high density lipoprotein; LDL – low density lipoprotein; FPG – fasting plasma glucose; R - correlation coefficient; p – statistical significance; * - statistical significant parameter.

In the group diagnosed with and treated for BPH, on the other hand, a positive correlation was only found in the group of patients without MetS, and it concerned the levels of propionic acid and triglycerides (R = 0.302, p = 0.024) (Table 6).

**Table 6:** Correlations between the analyzed SCFAs and biochemical parameters in patients with BPH without MetS and with MetS.

**Biochemical parameters**	**Patients with BPH without MetS (n=61)** **%SCFA**															
	**C2:0**		**C 3:0**		**C 4:0 i**		**C 4:0 n**		**C 5:0 i**		**C 5:0 n**		**C 6:0 i**		**C 6:0 n**	
	**R**	**p**	**R**	**p**	**R**	**p**	**R**	**p**	**R**	**p**	**R**	**p**	**R**	**p**	**R**	**p**
**TG** [mmol/l]	0.166	0.222	**0.302**	0.024*	-0.084	0.539	-0.138	0.312	-0.128	0.348	-0.143	0.294	0.139	0.306	-0.195	0.150
**Cholesterol** [mg/dl]	-0.084	0.534	0.225	0.093	-0.202	0.131	0.155	0.251	-0.201	0.134	-0.094	0.486	-0.215	0.108	-0.181	0.178
**HDL** [mg/dl]	-0.046	0.731	-0.180	0.180	0.111	0.409	0.064	0.637	0.120	0.372	0.130	0.335	-0.167	0.214	-0.033	0.810
**LDL** [mg/dl]	-0.113	0.410	0.163	0.234	-0.247	0.069	0.229	0.093	-0.241	0.076	-0.084	0.542	-0.245	0.072	-0.157	0.251
**FPG** [mg/dl]	-0.141	0.295	0.047	0.731	-0.146	0.279	0.111	0.411	-0.137	0.309	0.146	0.278	-0.205	0.126	0.154	0.253
**Biochemical parameters**	**Patients with BPH with MetS (n=42)** **%SCFA**															
	**C2:0**		**C 3:0**		**C 4:0 i**		**C 4:0 n**		**C 5:0 i**		**C 5:0 n**		**C 6:0 i**		**C 6:0 n**	
	**R**	**p**	**R**	**p**	**R**	**p**	**R**	**p**	**R**	**p**	**R**	**p**	**R**	**p**	**R**	**p**
**TG** [mmol/l]	-0.180	0.254	0.141	0.374	-0.009	0.955	0.055	0.728	-0.027	0.867	0.096	0.545	0.132	0.405	0.075	0.637
**Cholesterol** [mg/dl]	0.001	0.996	-0.098	0.538	-0.070	0.661	0.150	0.342	-0.129	0.417	-0.217	0.167	-0.030	0.852	-0.020	0.899
**HDL** [mg/dl]	0.034	0.833	0.130	0.412	-0.239	0.128	0.087	0.582	-0.185	0.241	-0.213	0.175	-0.166	0.294	-0.083	0.600
**LDL** [mg/dl]	0.028	0.860	-0.212	0.178	0.032	0.842	0.088	0.578	-0.039	0.808	-0.137	0.389	-0.143	0.367	0.009	0.957
**FPG** [mg/dl]	0.055	0.731	0.050	0.755	0.100	0.528	-0.079	0.618	0.084	0.596	-0.056	0.725	-0.293	0.060	-0.061	0.700

BPH – benign prostatic hyperplasia; MetS – metabolic syndrome; n – number; SCFAs – short-chain fatty acids; C2:0 - acetic acid; C3:0 - propionic acid; C4:0i - isobutyric acid; C4:0n - butyric acid; C5:0i - isovaleric acid; C5:0n - valeric acid; C6:0i - isocaproic acid; C 6:0n - caproic acid; TG – triglycerides; HDL – high density lipoprotein; LDL – low density lipoprotein; FPG – fasting plasma glucose; R - correlation coefficient; p – statistical significance, * - statistical significant parameter.

In healthy volunteers, depending on BMI, the percentage value of SCFAs negatively correlated with acetic acid (R = -0.221, p = 0.048) and positively with valeric acid (R = -0.287, p = 0.010), and in patients with BPH, it negatively correlated with isocaproic acid (R = -0.246, p = 0.014) (Supplementary Table 1). There were no statistically significant differences in BMI values between the patients. However, in the analysis of the correlation between SCFAs and MetS, this parameter was taken into account. In the entire study sample divided according to MetS, the presence of MetS negatively correlated with the level of propionic acid and BMI (R = -0.265, p = 0.020) (Supplementary Table 2).

In healthy patients without MetS, BMI did not correlate with SCFA levels, while in patients with MetS, it negatively correlated with propionic acid (C3: 0) (R = -0.517, p = 0.001) and positively with caproic acid (C6: 0n) (R = 0.329, p = 0.05) (Supplementary Table 3). In patients with BPH, no correlation between the levels of SCFAs and the presence of MetS was found (Supplementary Table 3).

## DISCUSSION

In our study, we indirectly examined the relationship between BPH, MetS and the intestinal microbiota, or more precisely its products - SCFAs. Analyzing the intestinal microbiota through testing its products is a relatively new method. The identification of SCFAs in the stool provides information not only about the composition of the intestinal microbiota, but also improves the understanding of how they interact not only in the gut but also in distant tissues and organs.

To the best of our knowledge, this is the first study investigating the association between the synthesis of SCFAs by the gut microbiota and BPH in aging men. The relationship between SCFAs and serum biochemical parameters in men with MetS have also been demonstrated. Our study showed that among the analyzed SCFAs, mainly isobutyric acid (C4:0i), isovaleric acid (C5:0i) and isocaproic acid (C6:0i) are those that very likely influence factors predisposing men to BPH.

We found that isobutyric acid was significantly elevated in men with BPH compared to healthy controls (*% SCFAs - mean: 4.695, median: 4.702 vs. 3.814, 3.726; p = 0.008*). The intestinal microbiota of BPH patients produced more isovaleric acid (*% SCFAs - mean: 9.499, median: 9.221 vs. 6.911, 6.730; p < 0.001*). The acid that predominated among the acids isolated from the feces of healthy controls was isocaproic acid (*% SCFAs - mean: 0.359, median: 0.174 vs. 0.186, 0.142; p = 0.038*).

SCFAs produced by gut microbiota - primarily acetate, propionate, butyrate play a key role in maintaining homeostasis in humans [7]. These three most common acids account for 95% in total SCFAs. In large intestine and stool samples SCFAs are present in an approximate molar ratio of acetic: propionic: butyric acid amounting to 60:20:20 [30]. The levels of SCFAs in the intestines range from 20 to 140 mM, and depend on the intestinal microflora composition, absorption of SCFAs from the intestines, and the fiber content in the diet [31]. Acetic, propionic and butyric acids are produced as a result of saccharolytic fermentation and has health promoting benefits. SCFAs are regarded as mediators in the communication between the intestinal microbiome and the immune system. The signal they produce is transferred, among others, in immune cells via free fatty acid receptors (FFARs), which belong to the family of G protein-coupled receptors (GPCRs) [32, 33]. It has been also confirmed that SCFAs inhibit the activity of histone deacetylase (HDAC) – an enzyme involved in post-translational modifications [9], namely the process of deacetylation and, what is new, the process of histone crotonylation [34]. These properties of SCFAs have an effect on their immunomodulatory potential i.e. maintaining the anti-proinflammatory balance. SCFAs act not only locally in the intestines colonized by commensal bacteria, but also influence the intestinal immune cells, and modulate immune response by multi-protein inflammasome complexes [35, 36]. Moreover, main SCFAs may affect fatty acids, glucose, and cholesterol metabolism.

The disturbances or changes in SCFAs levels may contribute to the development of many diseases. Currently conducted studies concern not only intestinal diseases, such as irritable bowel syndrome (IBS) [37], inflammatory bowel disease (IBD) [38], and diarrhea [39], but also mental health problems like depression [40], autism [41], neurodegenerative diseases (Parkinson’s disease) [14], multiple sclerosis [42], and even autoimmune diseases [43, 44]. The main SCFAs (acetate, propionate, butyrate) have immunomodulatory potential, and therefore may be helpful in the prevention of chronic but persistent low-grade inflammation. It is also worth noting that the concentrations of SCFAs and BCFAs fluctuate in a healthy population throughout life - from newborns to aging people [45]. It is influenced by: the composition of the intestinal microflora, age and health of patients. Many studies have confirmed that the intestinal microflora changes with the aging of the body [46, 47]. It turns out that the positive impact of microflora (characterized by a decrease in the taxonomic diversity of the intestinal microbiota) on the human body, decreases with age [48]. These changes are also reflected in the physiology of the host organism, and manifested as, among others, an increase in inflammation [49]. These differences are noted between adult men (mean age of 42 years) and elderly men (mean age of 77 years) [50]. In young people, bacteria that predominate in the composition of the intestinal microflora are those with immunomodulatory potential, such as *Clostridiales* and *Bifidobacterium*. In aging people, on the other hand, bacterial communities are enriched with pathobionts, e.g. *Enterobacteriaceae* [51].

Propionic acid (C3:0) is one of the main SCFAs. Its natural production takes place in the large intestine with the participation of *Bacteroidetes spp, Roseburia spp., Firmicutes, Roseburia inulinivorans, Ruminococus spp., Cllostridium spp., Clostridiales bactrium, Eubacterium spp, Coprococcus spp., Dialister succinatiphilus, Phascolarctobaterium succinatutens, Akkermansia muciniphila* (succinate pathway), *Clostridium sp., Clostridiales bacterium, Coproccus catus, Clostridium sp.,* (acrylate pathway), *Roseburia insulinivorans, Ruminococus spp., Eubacterium halli,* and *Clostridium sp.* (propanediol pathway) [52]. Propionic acid plays a role in the metabolism of lipids in the liver and affects the biosynthesis of cholesterol in this organ [53]. This acid is also used as a natural mold growth inhibitor and is therefore used as a preservative in food (cheese and bread) and animal feed. Moreover, it has been proven that propionic acid is a precursor in gluconeogenesis, while acetate and butyrate are involved in the regulation of cholesterol synthesis [54]. *In vivo* studies [55] have shown that propionic acid can inhibit de novo fatty acids and cholesterol. In addition, propionic acid has been shown to significantly reduce the levels of pro-inflammatory cytokines (TNF-α and CCL5), and increase the expression of lipoprotein lipase and GLUT4, thus influencing the lipogenesis process and glucose uptake [56]. It has been also confirmed that propionate significantly stimulates the production of peptides in the large intestine: peptide YY (PYY) and glucagon like peptide-1 (GLP-1), which are responsible for the regulation of appetite in adults, thus reducing the amount of food intake and weight gain in obese people [57].

Butyric acid (C4:0) is an important factor influencing the metabolic processes, which has been confirmed both in animal models and in humans, yet the exact mechanism of its action still requires a more detailed explanation. It has been proven that butyric acid supplementation prevents obesity, hyperinsulinemia and hypertriglyceridemia, and may also reduce appetite and activate brown adipose tissue (BAT) through vagal nerve signaling [58]. Butyric and valeric acids have also been shown to be class I histone deacetylase inhibitors [59].

There is also little information about the effects of caproic acid (C6:0) or isocaproic acid (C6:0i) on BPH. In the research conducted so far, the impact of caproic acid on prostate cancer cells has been confirmed in the studies conducted so far on cell lines. The C6 acid has been shown to have cytotoxic properties against neoplastic cells [60] Studies on a rat model, on the other hand, showed that in the feces of animals with nonbacterial chronic prostate inflammation (CPI), the levels of butyric, valeric, and caproic SCFAs were decreased. This study also indicates that prostate inflammation is associated with specific changes in the gut microflora, and hence with changes in SCFA levels. [61]. Additionally, it can be mentioned that caproic acid is one of the compounds found in saw palmetto, which is used in natural BPH therapy [62].

Branched SCFAs — branched short-chain fatty acids (BCFAs), such as, isobutyric acid (C4:0i) and isovaleric acid (C5:0i), which are the end-products of aliphatic amino acid catabolism, valine and leucine respectively [63]. Isobutyric acid (C4:0i) is a geometrical isomer of butyric acid, which results in its different physical but not chemical properties. In the human intestine, the fermentation of branched chain amino acids is carried out mainly by genera *Bacteroides* and *Clostridium* [64]. It has also been found that gut-derived BCFAs - mainly isovaleric acid, may be one of the contributors to depressive disorders [65], or even be associated with the occurrence of acute ischemic stroke [66]. Among the bacterial populations that participate in the fermentation of peptides and amino acids, there are also those bacteria that are responsible for the production of BCFAs—0.6% of the population is involved in the synthesis of isovaleric acid, and up to 40% of bacteria in the synthesis of isobutyric acid [67]. The highest levels of BCFAs are found in the distal parts of the large intestine (colon). Among short-chain fatty acids, branched forms account for 5-10%. The concentrations of BCFAs in the feces, as well as the concentrations of SCFAs, may be modified depending on the food consumed [68]. Little is known so far about the effects of BCFAs on the host organism. However, there is evidence that these acids can oxidize if the amount of butyric acid is insufficient and then they can be a source of energy for colonocytes [69].

**Figure 1:**
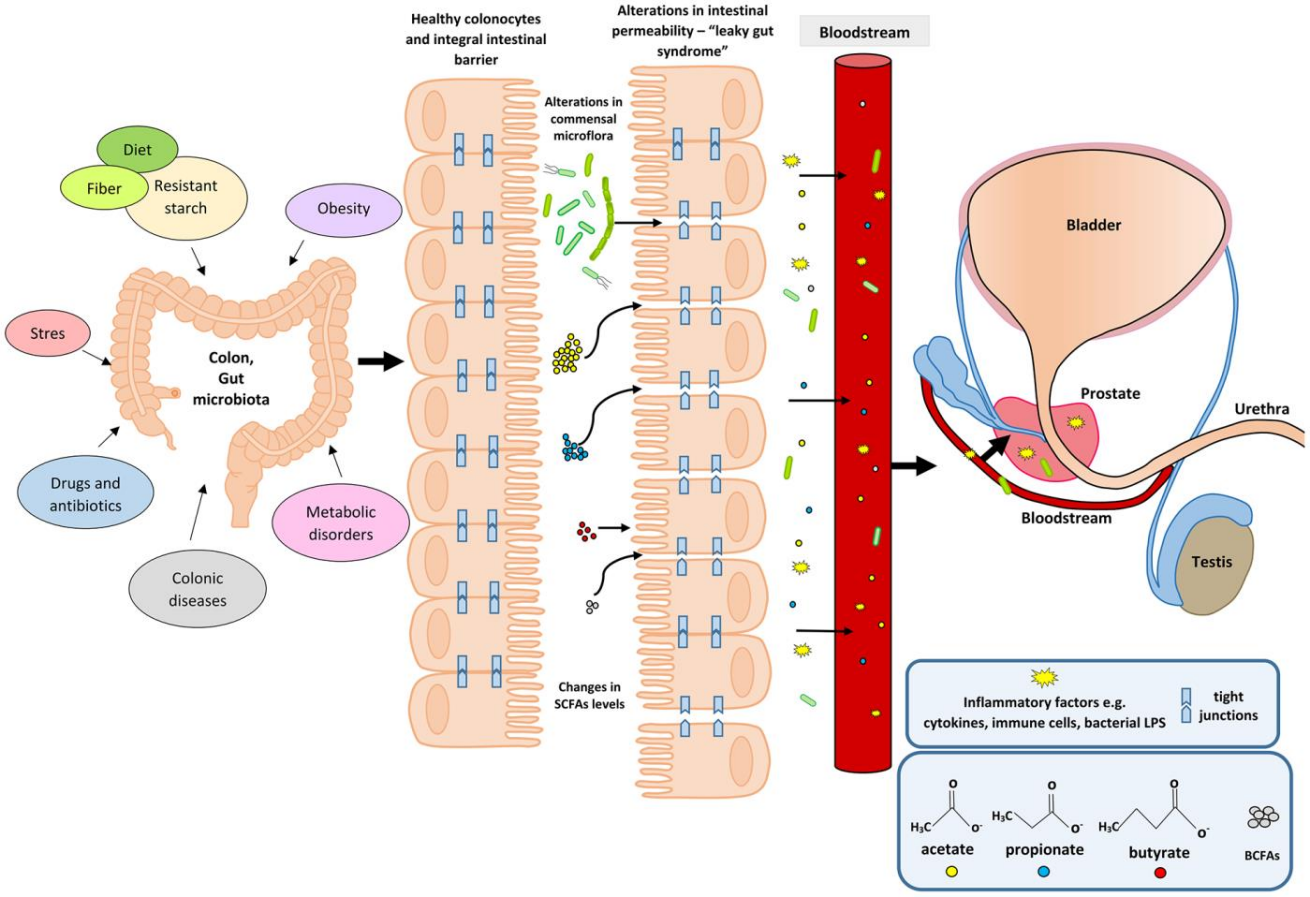
**Participation of the intestinal microflora in inflammation and the development of BPH.** Disturbances in the intestinal microflora (dysbiosis) can be caused by many factors, among them bad diet that is poor in plant fiber and starch sources. Additionally, the state of intestinal eubiosis may be disturbed by taking antibiotics and other medications, e.g. in the treatment of diabetes or depressive disorders. The specific intestinal microflora—‘obese microbiota’—is found in overweight people and it additionally affects metabolic processes in these people. A different gut microbiome is also observed in people with inflammatory bowel diseases (IBS, IBD). Proper intestinal microflora and its metabolites produced in the fermentation process, including short-chain fatty acids (SCFAs) contribute to the maintenance of intestinal epithelial cell homeostasis, and are a source of energy for colonocytes (mainly butyric acid). Intestinal dysbiosis and changes in SCFAs levels are factors that reduce the protective mucus layer, weaken tight junctions between intestinal epithelial cells, and cause leakage of the intestinal barrier. When the intestinal barrier is disturbed, pathogenic factors, inflammatory factors (immune cells and cytokines) and bacterial metabolites produced in varying amounts, e.g. SCFAs and toxic metabolites enter the bloodstream and migrate to distant tissues and organs. Inflammatory and microbiological factors, along with the peripheral circulation, may also reach the prostate gland, where they cause local inflammation. The inflammatory process in the prostate can activate signaling pathways involving growth factors, thus resulting in the prostate proliferation.

An upsurge in the amount of BCFAs may indicate an increased proteolytic fermentation, which suggests that there is a higher amount of proteins in the large intestine. This in turn may be caused by a high supply of protein in the diet or its disturbed absorption. Moreover, during proteolytic fermentation, along with the increase in BCFAs, harmful metabolites are produced such as: ammonia, phenols and hydrogen sulphides [67]. The resulting components may contribute to the initiation of inflammation and the excessive proliferation of colonocytes, and thus influence the local disease states. Apart from inducing epithelial cell proliferation, inflammation may also affect tight junctions (TJ). This happens in the case of infections (including *Helicobakter pylorii*) and gastric epithelial disruption [70]. Damage to tight junctions results in the leakage and dysfunction of cellular barriers, and is the cause of ‘leaky states’ and chronic inflammation, which are observed in cancers of the digestive system [71, 72]. Impairment of the function of tight junction in the digestive tract (mainly in the intestines) may also be caused by the state of intestinal dysbiosis and reduced amounts of SCFAs, resulting, among others, from using antibiotics [73]. SCFAs, mainly butyric acid, strengthen the intestinal barrier by regulating the transcription of claudin -1 that is a protein building tight junctions [74]. The leakage of the intestinal barrier results in the penetration of bacterial particles and factors, pro-inflammatory cytokines, immune cells, toxins and antigens into the bloodstream and their movement from the digestive tract to distant places, including the prostate [75] (Figure 1).

Many studies have confirmed that SCFAs are involved in the pathophysiology of IBD and may be a prognostic marker of the disease state [76]. Jaworska et al. [76] demonstrated that compared with healthy individuals, the ratio of acids in serum to acids in feces (acetate, valerate, isocaproic, caproic and propionic acids) is statistically significantly higher in patients with IBD. These data indicate that SCFAs may be involved in the disturbance of the intestinal barrier function [76]. The research by Huda-Jaujan et al. [38] also confirmed the role of SCFAs in the pathogenesis of IBD. Their analysis showed that in people with inflammatory bowel disease, the levels of the main fatty acids—acetic, butyric and propionic— drop dramatically compared to healthy people (162.0, 86.9, 65.6 μmol/g vs. 209.7, 176.0, 93.3 μmol/g) [38]. Tian et al. [37] reported that serum levels of propionic and butyric acids are increased in diarrhea-predominant IBS (IBS-D) patients compared to healthy controls. Such a dependence was not observed in relation to SCFA levels in patients’ feces. These data indicate that elevated acid levels may influence the development of IBS-D [37]. A meta-analysis of studies on IBS patients showed [77] that the levels of propionic acid in their stools were significantly higher than in the feces of healthy people. Additionally, butyric acid predominated in patients with IBS-D type [77]. The study also confirmed statistically significant differences in the percentages of propionic acid (20.20 vs. 17.85, p = 0.007) and butyric acid (15.58 vs. 19.00, p = 0.003) in the stool samples between patients with IBS and healthy individuals, which indicates that SCFAs are good, non-invasive markers of gut disease [78].

Changes in fecal short-chain fatty acids have also been observed in patients with morbid obesity after surgical interventions. As they lost weight and changed their diet, the total amount of SCFAs decreased. A similar effect was obtained for the relative amounts of straight chain SCFAs, namely acetic, propionic and butyric acids. At the same time, the levels of BCFAs (isobutyric, isovaleric, and isocaproic acids) increased [79]. These changes suggest predominant proteolytic fermentation that may have adverse health effects. A therapy may be to change a high-protein diet to a diet rich in carbohydrates, fiber, and polysaccharides in order to increase saccharolytic fermentation and the level of main straight SCFAs (acetate, propionate, and butyrate), having health-promoting properties [79].

The effect of the intestinal microflora and its metabolism products (SCFAs, including BCFAs) on BPH has not been studied so far. However, there are studies showing that IBD may affect prostate disease and increase the risk of prostate cancer [19]. In the pilot study by Golombos et al. [20], differences in the composition of the intestinal microflora were observed between patients with BPH and PCa, which may indicate the role of the intestinal microflora in the pathogenesis of prostate diseases. In the study by Liss et al. [17], fecal microbiome of men with and without prostate cancer was analyzed. There were no differences in the species composition of bacteria between the studied groups—so far no ‘intestinal microbiological profile’ predisposing to cancer development has been found. However, it has been noticed that in patients diagnosed with PCa, the natural production of vitamin B and folic acid by intestinal bacteria is disturbed [17]. Moreover, long-term intestinal inflammation may increase circulating pro-inflammatory cytokines, which may also contribute to inflammation in the prostate. It has already been confirmed that chronic inflammation in the body, not always associated with microbial infection of the genitourinary system in men, but resulting from an excess of adipose tissue and MetS, can contribute to the development of PCa [80]. Chronic inflammation is a common etiological factor for both BPH [81–84] and PCa [23, 85–88]. Previous studies [18] indicate differences in the composition of the intestinal microflora between healthy individuals and patients with prostate cancer treated with androgen axis-targeted therapies. It has been found that oral hormone therapy for prostate cancer may disrupt the normal intestinal microflora and additionally affect the clinical response of patients to other therapies, including immunological ones [18].

In our study, we also observed a statistically significant relationship between the percentage of some SCFAs—propionic acid (C3: 0, C5: 0n, and C6: 0n), and the presence of MetS, both in the control group and patients with BPH. Patients with BPH and MetS had significantly higher stool BCFA levels— isobutyric acid (p = 0.044) and isovaleric acid (p = 0.029) and lower isocaproic acid (p = 0.019) compared to healthy controls. We showed no differences in the amount of C4: 0. This is due to the fact that butyric acid is used very quickly by intestinal epithelial colonocytes as a source of energy. We found a significant correlation between biochemical parameters and SCFA levels in men without BPH and with MetS— triglycerides and cholesterol, and propionic acid *(R = 0.331, p = 0.049; R = 0.399, p = 0.016)*, and in patients with BPH and without MetS— triglycerides and propionic acid *(R = 0.302, p = 0.024)*. In addition, in patients without BPH, irrespective of MetS, propionic acid correlated with triglycerides, cholesterol, and low-density lipoproteins (LDL) (*R = 0.385, p < 0.005; R = 0.290, p = 0.010; R = 0.244, p = 0.030*).

There are many studies showing that the intestinal microflora and the bacterial metabolites it produces are involved in the metabolic processes in the body. There is also an association between intestinal inflammation and MetS [89]. Studies carried out in animal models indicate that the presence of microorganisms inhabiting the intestines in the population, e.g. the *Lactobacillus (L.) rhamnosus BFE5264* strain, increases the levels of propionate in the blood serum of animals. Moreover, these changes are accompanied by lowering cholesterol levels. These data suggest that both the respective bacterial strains and the metabolites produced with their participation (e.g. SCFAs) may influence the biosynthesis of cholesterol. It is worth noting that the tested *L. rhamnosus BFE5264* strain comes from Maasai fermented milk consumed by this social group, which, despite a diet rich in animal fats, has low blood serum cholesterol levels [90]. Tirosh et al. [91] proved that propionate, both in mice and in humans, contributes to metabolic disorders and may even cause gradual weight gain. Propionate, in too high a concentration, via catecholamines (insulin antagonists), can activate signaling pathways that lead to an increase in hepatic glucose production. It can also reduce its uptake and use by peripheral tissues, which in turn may lead to insulin resistance and hyperinsulinemia [91]. In turn, other studies have shown that BCFAs - mainly isobutyric acid, significantly increased glucose uptake and may contribute to increased insulin sensitivity in people with disorders of its metabolism, which was also shown for propionic acid [92]. Research by Granado-Serrano et al. [93] showed that in patients with hypercholesterolaemia, the SCFAs profile isolated from patients' faeces were dominated by: isobutyric and isovaleric acid. Moreover, isobutyric acid positively correlated with *Odoribacter* and blood lipid parameters. Further research is needed in order to elucidate the mechanisms of action of BCFA in health and disease.

In a prospective cohort study [94] on the Danish population (893 participants), 34 bacterial taxa related to BMI and blood lipids were identified. It was shown that, irrespective of age, sex, and genetic factors, the intestinal microbiota can affect BMI, triglycerides and high-density lipoproteins (HDL), and have a slight impact on low-density lipoproteins (LDL) and total cholesterol [94]. These data indicate that the intestinal microflora, including its metabolites, may be a potential therapeutic target in obesity and lipid disorders, which are components of MetS contributing to the development of BPH. The appropriate proportions and concentrations of SCFAs, produced by the microbiota, affect the homeostasis of metabolism, and therefore may help prevent MetS and type II diabetes [94]. It has also been confirmed that propionic acid supplementation in adults significantly reduces weight gain. It also affects the distribution of abdominal adipose tissue and reduces the content of lipids inside the liver cells in people without non-alcoholic fatty liver disease, and inhibits the development of insulin sensitivity [57]. Research by Bouter et al. [54] also indicate a positive contribution of SCFAs (the study analyzed butyrate) on glucose metabolism in lean people. Propionate produced by the gut microflora has also been confirmed to correlate with a reduced likelihood of developing MetS and its more effective treatment, as well as obesity-related diseases. This is a direct, anti-inflammatory effect of C3: 0 acid on the visceral adipose tissue and an increase in lipogenesis and glucose uptake [95]. Other studies [96], also conducted on humans, reveal differences in SCFAs levels between obese and lean people. In overweight people, the levels of acetate, propionate, butyrate, valerate, and total SCFAs are higher than in lean participants, but no differences have been found between the identified bacterial strains. The available data confirm the hypothesis that between obese and lean people, despite the lack of nutritional differences, colonic fermentation is different, which reflects the presence of the ‘obese microbiome’, and contributes to the observed changes in SCFAs levels [96]. The diet and the composition of the intestinal microflora have a significant effect on the quantitative and qualitative composition of the produced metabolites, including SCFAs, and thus on the development of MetS. Studies in which adult patients diagnosed with MetS were subjected to dietary interventions have confirmed this thesis [57, 97, 98]. Dietary interventions involved the supply of food with a high level of polysaccharides modifying the composition of the intestinal microflora, and increasing the production of SCFAs, mainly acetate and butyrate [97]. The authors of this study also noted that a proper diet significantly reduces the levels of BCFAs (isobutyrate and isovalerate), which additionally indicates a reduction in protein fermentation and the production and accumulation of metabolites damaging the intestinal epithelium and cellular connections [97]. The bacteria predominating in the intestinal microflora of lean people are specific, probiotic bacteria, namely *Bifidobacterium* and *Akkermansia muciniphila*, producing health-promoting SCFAs—they, among others, increase insulin sensitivity and maintain the intestinal barrier, which supports the homeostasis of intestinal cells and reduces local and systemic inflammation [99].

## CONCLUSIONS

We explored variations of SCFAs in fecal samples from healthy controls and patients with BPH. We observed increased levels of isobutyric acid (C4:0i) and isovaleric acid (C5:0i) in the feces of patients with BPH in this study. The gut microbiota is very likely to be indirectly involved in the development of BPH through isobutyric and isovaleric acid. SCFAs associated with BPH would be a useful focus for future studies. Due to the limitations of the present study, further investigation into SCFAs in BPH patients, and a probe of gut microbiota is warranted. The differences between isobutyric acid (C4:0i), isovaleric acid (C5:0i) and isocaproic acid (C6:0i) were associated with the occurrence of MetS in patients. Moreover, it was shown that propionic acid was found to be related to MetS and its selected diagnostic parameters (blood lipid levels) in healthy control patients.

The obtained data indicate that SCFAs produced by intestinal bacteria participate in the development of MetS in patients with BPH. Moreover, the tested SCFAs may also be involved in the modulation of biochemical parameters diagnostic for MetS, but their exact mechanism of action is still not entirely clear. They also show that in the intestines of BPH patients, proteolytic fermentation occurs, resulting in the formation of BCFAs. Previous studies, in patients with IBD, indicate that the production of BCFAs is accompanied by the production of harmful metabolites that contribute to the damage of the intestinal barrier. In turn, the disturbed intestinal barrier in patients with BPH may lead to the penetration of bacterial and inflammatory factors into the systemic circulation, which may result in the prostate gland inflammation and induction of growth factors responsible for the proliferation of prostate cells.

However, further research is needed to confirm our results and thesis.

## MATERIALS AND METHODS

### Patients

The study involved 103 men diagnosed with and treated for BPH, who had undergone transurethral resection of the prostate (TURP) at the Clinic of Urology and Urological Oncology, Pomeranian Medical University, Szczecin, Poland between November 2017 and May 2019. The men were aged 49–79 years (mean age: 66.4). The diagnosis was based on the results of the International Prostate Symptom Score (IPSS) questionnaire (with one question relating to overall quality of life - QoL), long lasting symptoms, decreased flow rate (Q_max_) or urinary retention, and increased prostate volume. BPH was confirmed in prostate tissue removed from the prostate gland during the TURP procedure.

The control group included healthy volunteers (n = 80) aged 45-72 years (mean: 54.7) with prostate size ≤ 30 ml and PSA less than 2.5 ng/ml. In this group, the IPSS questionnaire scores did not exceed seven points, the patients did not report any symptoms of BPH. Healthy volunteers were enrolled in this study between December 2019 and October 2019.

The criteria for exclusion from the study were: active cancer disease, alcoholism, thyroid diseases, taking glucocorticosteroids and antibiotics for six months preceding the examination. Only those patients from whom we obtained a complete set of material for laboratory tests (serum and stool sample) were included in the research. The study was approved by the Bioethical Commission of the Pomeranian Medical University, Szczecin (approval number KB-0012/139/17). All participants gave their informed written consent to take part in the study.

### Clinical examination

Anthropometric measurements were performed in all patients—body weight, height and waist circumference. The participants of the study completed questionnaires concerning demographic data and health status. Based on the criteria presented by the International Diabetes Federation (IDF) in 2005 [100], the men were divided according to the presence of MetS. The men with abdominal obesity were qualified for the MetS group if they had waist circumference ≥ 94 cm and at least two of the following abnormalities: triglycerides (TG) ≥ 150 mg/dl or treatment for dyslipidemia; high-density lipoprotein (HDL) cholesterol < 40 mg/dl or treatment for dyslipidemia*;* fasting glycemia ≥ 100 mg/dl or treatment for type 2 diabetes; blood pressure ≥ 130/85 mmHg or treatment for hypertension. All components of MetS were considered both individually and as sets of symptoms.

### Blood serum analysis

To evaluate basic biochemical parameters, such as the serum levels of glucose, total cholesterol, low-density lipoprotein cholesterol (LDL), high-density lipoprotein cholesterol (HDL), triglycerides (TG), we collected blood using the Vacutainer system tubes with clot activator and gel separator. 7.5 ml blood samples were taken from a cubital vein on an empty stomach between 7.30 am and 9.00 am. The blood was collected by qualified medical staff and delivered to the laboratory in accordance with the relevant rules and procedures. The parameters were determined using a spectrophotometric method with commercial reagent kits.

### Stool sampling

Patients from study and control group was asked to collect a stool sample into a screw-capped collection container using a plastic holder to use the collection container in the toilet. Study participants were advised not to use laxatives. Study participants were sampling feces after overnight fasting. After the stool was collected, patients were delivering the samples within 24 h to our laboratory. The samples were then stored at −80° C until the analyses.

### Short-chain fatty acids

### Isolation of short-chain fatty acids

Isolation was performed by suspending 0.5 g of a stool sample in a test tube with 2.5 ml of deionized water. The samples were thoroughly mixed on a shaker for 10 minutes. The pH of the samples was then checked and brought to a final pH of 2 to 3 by the addition of 200 μl of 2M HCl. 36 μl of the internal standard (IS) was added to each sample. The samples were then centrifuged for 10 minutes at 5000 rpm. Next, the obtained supernatant was transferred to vials through a syringe filter (Ø 400 μm) and analyzed by gas chromatography.

### Gas chromatography

Gas chromatographic analysis was performed using an Agilent Technologies 1260 A GC chromatograph and a flame ionization detector (FID). A fused silica (quartz) capillary column with a free fatty acid phase (DB-FFAP, 30 m × 0.53 mm × 0.5 μm) was used for the analyzes. The carrier gas (mobile phase) was hydrogen at a flow rate of 14.4 ml / min. The analysis was done with a temperature gradient, the starting temperature was 100° C and was held for 0.5 minutes. The temperature was then increased by another 8° C and held for 1 minute until the temperature was 180° C. Eventually the temperature was increased to 200° C by increasing it by 20° C for a minute and then keeping it for 5 minutes. The injection volume of one sample was 1 μl, and the analysis time for one sample was 17.5 minutes. The following SCFAs were analyzed in the study: acetic acid (C2:0), propionic acid (C3:0), isobutyric acid (C4:0i), butyric acid (C4:0 n), isovaleric acid (C5:0i), valeric acid (C5:0n), isocaproic acid (C6:0i), caproic acid (C6:0n), and enanthic acid (C7:0). However, in the analysis of the results, C2:0 - C6:0 acids (produced with the participation of the intestinal microflora) were taken into account.

### Statistical analysis

Statistical analysis was performed using the SPSS Statistics 17.0 software. The study sample was described in terms of basic statistics (mean, standard deviation (SD), minimum, and maximum values). The normality of the distribution was assessed using the Shapiro–Wilk test. The differences between the groups were determined by the Mann–Whitney U test. Spearman’s rank correlation coefficient was applied. The level of significance was set at p ≤ 0.05.

### Limitations of the study

One of the limitations of the study is the fact that there is an age difference between the study group and the control group, however, both groups include aging men. In our study, we only analyzed the short-chain fatty acids qualitatively and quantitatively. Another limitation is that our research did not analyze the patients’ diet and the composition of their intestinal microflora, but we plan to analyze these aspects in our future studies. What is important, we excluded from the study patients who used antibiotic therapy, antidepressants or glucocorticosteroids, which are factors disrupting the intestinal microflora, within six months prior to the start of the study. The results of our research motivate us to carry out further analyzes, taking into account new variables, to make the research more precise.

## Supplementary Material

Supplementary Tables

## References

[1] Parsons JK. Benign Prostatic Hyperplasia and Male Lower Urinary Tract Symptoms: Epidemiology and Risk Factors. Curr Bladder Dysfunct Rep. 2010; 5:212–18. 10.1007/s11884-010-0067-2 21475707 PMC3061630

[2] Gacci M, Sebastianelli A, Salvi M, De Nunzio C, Vignozzi L, Corona G, Jaeger T, Chini T, Russo GI, Maggi M, Morgia G, Tubaro A, Carini M, Serni S. Benign prostatic enlargement can be influenced by metabolic profile: results of a multicenter prospective study. BMC Urol. 2017; 17:22. 10.1186/s12894-017-0211-9 28376747 PMC5379726

[3] Park YW, Kim SB, Kwon H, Kang HC, Cho K, Lee KI, Kim YJ, Lee JH. The relationship between lower urinary tract symptoms/benign prostatic hyperplasia and the number of components of metabolic syndrome. Urology. 2013; 82:674–79. 10.1016/j.urology.2013.03.047 23850334

[4] Zhao S, Chen C, Chen Z, Xia M, Tang J, Shao S, Yan Y. Relationship between Metabolic Syndrome and Predictors for Clinical Benign Prostatic Hyperplasia Progression and International Prostate Symptom Score in Patients with Moderate to Severe Lower Urinary Tract Symptoms. Urol J. 2016; 13:2717–26. 27351328

[5] Gacci M, Vignozzi L, Sebastianelli A, Salvi M, Giannessi C, De Nunzio C, Tubaro A, Corona G, Rastrelli G, Santi R, Nesi G, Serni S, Carini M, Maggi M. Metabolic syndrome and lower urinary tract symptoms: the role of inflammation. Prostate Cancer Prostatic Dis. 2013; 16:101–06. 10.1038/pcan.2012.44 23165431

[6] Vignozzi L, Gacci M, Maggi M. Lower urinary tract symptoms, benign prostatic hyperplasia and metabolic syndrome. Nat Rev Urol. 2016; 13:108–19. 10.1038/nrurol.2015.301 26754190

[7] Tramontano M, Andrejev S, Pruteanu M, Klünemann M, Kuhn M, Galardini M, Jouhten P, Zelezniak A, Zeller G, Bork P, Typas A, Patil KR. Nutritional preferences of human gut bacteria reveal their metabolic idiosyncrasies. Nat Microbiol. 2018; 3:514–22. 10.1038/s41564-018-0123-9 29556107

[8] Pryde SE, Duncan SH, Hold GL, Stewart CS, Flint HJ. The microbiology of butyrate formation in the human colon. FEMS Microbiol Lett. 2002; 217:133–39. 10.1111/j.1574-6968.2002.tb11467.x 12480096

[9] Grabarska A, Dmoszyńska-Graniczka M, Nowosadzka E, Stepulak A. [Histone deacetylase inhibitors - molecular mechanisms of actions and clinical applications]. Postepy Hig Med Dosw (Online). 2013; 67:722–35. 10.5604/17322693.1061381 24018438

[10] Milligan G, Shimpukade B, Ulven T, Hudson BD. Complex Pharmacology of Free Fatty Acid Receptors. Chem Rev. 2017; 117:67–110. 10.1021/acs.chemrev.6b00056 27299848

[11] Park J, Kim M, Kang SG, Jannasch AH, Cooper B, Patterson J, Kim CH. Short-chain fatty acids induce both effector and regulatory T cells by suppression of histone deacetylases and regulation of the mTOR-S6K pathway. Mucosal Immunol. 2015; 8:80–93. 10.1038/mi.2014.44 24917457 PMC4263689

[12] den Besten G, van Eunen K, Groen AK, Venema K, Reijngoud DJ, Bakker BM. The role of short-chain fatty acids in the interplay between diet, gut microbiota, and host energy metabolism. J Lipid Res. 2013; 54:2325–40. 10.1194/jlr.R036012 23821742 PMC3735932

[13] Barrea L, Muscogiuri G, Annunziata G, Laudisio D, Pugliese G, Salzano C, Colao A, Savastano S. From gut microbiota dysfunction to obesity: could short-chain fatty acids stop this dangerous course? Hormones (Athens). 2019; 18:245–50. 10.1007/s42000-019-00100-0 30840230

[14] Aho VT, Houser MC, Pereira PA, Chang J, Rudi K, Paulin L, Hertzberg V, Auvinen P, Tansey MG, Scheperjans F. Relationships of gut microbiota, short-chain fatty acids, inflammation, and the gut barrier in Parkinson’s disease. Mol Neurodegener. 2021; 16:6. 10.1186/s13024-021-00427-6 33557896 PMC7869249

[15] Morrison DJ, Preston T. Formation of short chain fatty acids by the gut microbiota and their impact on human metabolism. Gut Microbes. 2016; 7:189–200. 10.1080/19490976.2015.1134082 26963409 PMC4939913

[16] Eberhart BL 2nd, Wilson AS, O’Keefe SJ, Ramaboli MC, Nesengani LT. A simplified method for the quantitation of short-chain fatty acids in human stool. Anal Biochem. 2021; 612:114016. 10.1016/j.ab.2020.114016 33188741

[17] Liss MA, White JR, Goros M, Gelfond J, Leach R, Johnson-Pais T, Lai Z, Rourke E, Basler J, Ankerst D, Shah DP. Metabolic Biosynthesis Pathways Identified from Fecal Microbiome Associated with Prostate Cancer. Eur Urol. 2018; 74:575–82. 10.1016/j.eururo.2018.06.033 30007819 PMC6716160

[18] Sfanos KS, Markowski MC, Peiffer LB, Ernst SE, White JR, Pienta KJ, Antonarakis ES, Ross AE. Compositional differences in gastrointestinal microbiota in prostate cancer patients treated with androgen axis-targeted therapies. Prostate Cancer Prostatic Dis. 2018; 21:539–48. 10.1038/s41391-018-0061-x 29988102 PMC6283851

[19] Burns JA, Weiner AB, Catalona WJ, Li EV, Schaeffer EM, Hanauer SB, Strong S, Burns J, Hussain MH, Kundu SD. Inflammatory Bowel Disease and the Risk of Prostate Cancer. Eur Urol. 2019; 75:846–52. 10.1016/j.eururo.2018.11.039 30528221 PMC6542355

[20] Golombos DM, Ayangbesan A, O’Malley P, Lewicki P, Barlow L, Barbieri CE, Chan C, DuLong C, Abu-Ali G, Huttenhower C, Scherr DS. The Role of Gut Microbiome in the Pathogenesis of Prostate Cancer: A Prospective, Pilot Study. Urology. 2018; 111:122–28. 10.1016/j.urology.2017.08.039 28888753

[21] Shah A, Shah AA, K N, Lobo R. Mechanistic targets for BPH and prostate cancer-a review. Rev Environ Health. 2020. [Epub ahead of print]. 10.1515/reveh-2020-0051 32960781

[22] Miah S, Catto J. BPH and prostate cancer risk. Indian J Urol. 2014; 30:214–18. 10.4103/0970-1591.126909 24744523 PMC3989826

[23] De Marzo AM, Platz EA, Sutcliffe S, Xu J, Grönberg H, Drake CG, Nakai Y, Isaacs WB, Nelson WG. Inflammation in prostate carcinogenesis. Nat Rev Cancer. 2007; 7:256–69. 10.1038/nrc2090 17384581 PMC3552388

[24] Kramer G, Mitteregger D, Marberger M. Is benign prostatic hyperplasia (BPH) an immune inflammatory disease? Eur Urol. 2007; 51:1202–16. 10.1016/j.eururo.2006.12.011 17182170

[25] Gandaglia G, Briganti A, Gontero P, Mondaini N, Novara G, Salonia A, Sciarra A, Montorsi F. The role of chronic prostatic inflammation in the pathogenesis and progression of benign prostatic hyperplasia (BPH). BJU Int. 2013; 112:432–41. 10.1111/bju.12118 23650937

[26] Park J, Goergen CJ, HogenEsch H, Kim CH. Chronically Elevated Levels of Short-Chain Fatty Acids Induce T Cell-Mediated Ureteritis and Hydronephrosis. J Immunol. 2016; 196:2388–400. 10.4049/jimmunol.1502046 26819206 PMC4761537

[27] Moser AM, Spindelboeck W, Strohmaier H, Enzinger C, Gattringer T, Fuchs S, Fazekas F, Gorkiewicz G, Wurm P, Högenauer C, Khalil M. Mucosal biopsy shows immunologic changes of the colon in patients with early MS. Neurol Neuroimmunol Neuroinflamm. 2017; 4:e362. 10.1212/NXI.0000000000000362 28638851 PMC5471347

[28] Cait A, Hughes MR, Antignano F, Cait J, Dimitriu PA, Maas KR, Reynolds LA, Hacker L, Mohr J, Finlay BB, Zaph C, McNagny KM, Mohn WW. Microbiome-driven allergic lung inflammation is ameliorated by short-chain fatty acids. Mucosal Immunol. 2018; 11:785–95. 10.1038/mi.2017.75 29067994

[29] Zhang X, Chen BD, Zhao LD, Li H. The Gut Microbiota: Emerging Evidence in Autoimmune Diseases. Trends Mol Med. 2020; 26:862–73. 10.1016/j.molmed.2020.04.001 32402849

[30] Hijova E, Chmelarova A. Short chain fatty acids and colonic health. Bratisl Lek Listy. 2007; 108:354–58. 18203540

[31] Rooks MG, Garrett WS. Gut microbiota, metabolites and host immunity. Nat Rev Immunol. 2016; 16:341–52. 10.1038/nri.2016.42 27231050 PMC5541232

[32] Bolognini D, Tobin AB, Milligan G, Moss CE. The Pharmacology and Function of Receptors for Short-Chain Fatty Acids. Mol Pharmacol. 2016; 89:388–98. 10.1124/mol.115.102301 26719580

[33] Ohira H, Tsutsui W, Fujioka Y. Are Short Chain Fatty Acids in Gut Microbiota Defensive Players for Inflammation and Atherosclerosis? J Atheroscler Thromb. 2017; 24:660–72. 10.5551/jat.RV17006 28552897 PMC5517538

[34] Fellows R, Denizot J, Stellato C, Cuomo A, Jain P, Stoyanova E, Balázsi S, Hajnády Z, Liebert A, Kazakevych J, Blackburn H, Corrêa RO, Fachi JL, et al. Microbiota derived short chain fatty acids promote histone crotonylation in the colon through histone deacetylases. Nat Commun. 2018; 9:105. 10.1038/s41467-017-02651-5 29317660 PMC5760624

[35] Levy M, Thaiss CA, Zeevi D, Dohnalová L, Zilberman-Schapira G, Mahdi JA, David E, Savidor A, Korem T, Herzig Y, Pevsner-Fischer M, Shapiro H, Christ A, et al. Microbiota-Modulated Metabolites Shape the Intestinal Microenvironment by Regulating NLRP6 Inflammasome Signaling. Cell. 2015; 163:1428–43. 10.1016/j.cell.2015.10.048 26638072 PMC5665753

[36] Yuan X, Wang L, Bhat OM, Lohner H, Li PL. Differential effects of short chain fatty acids on endothelial Nlrp3 inflammasome activation and neointima formation: Antioxidant action of butyrate. Redox Biol. 2018; 16:21–31. 10.1016/j.redox.2018.02.007 29475132 PMC5842312

[37] Tian Z, Zhuang X, Luo M, Yin W, Xiong L. The propionic acid and butyric acid in serum but not in feces are increased in patients with diarrhea-predominant irritable bowel syndrome. BMC Gastroenterol. 2020; 20:73. 10.1186/s12876-020-01212-3 32178625 PMC7077160

[38] Huda-Faujan N, Abdulamir AS, Fatimah AB, Anas OM, Shuhaimi M, Yazid AM, Loong YY. The impact of the level of the intestinal short chain Fatty acids in inflammatory bowel disease patients versus healthy subjects. Open Biochem J. 2010; 4:53–58. 10.2174/1874091X01004010053 20563285 PMC2887640

[39] Ramakrishna BS, Mathan VI. Colonic dysfunction in acute diarrhoea: the role of luminal short chain fatty acids. Gut. 1993; 34:1215–18. 10.1136/gut.34.9.1215 8406157 PMC1375457

[40] Skonieczna-Żydecka K, Grochans E, Maciejewska D, Szkup M, Schneider-Matyka D, Jurczak A, Łoniewski I, Kaczmarczyk M, Marlicz W, Czerwińska-Rogowska M, Pełka-Wysiecka J, Dec K, Stachowska E. Faecal Short Chain Fatty Acids Profile is Changed in Polish Depressive Women. Nutrients. 2018; 10:1939. 10.3390/nu10121939 30544489 PMC6316414

[41] Liu S, Li E, Sun Z, Fu D, Duan G, Jiang M, Yu Y, Mei L, Yang P, Tang Y, Zheng P. Altered gut microbiota and short chain fatty acids in Chinese children with autism spectrum disorder. Sci Rep. 2019; 9:287. 10.1038/s41598-018-36430-z 30670726 PMC6342986

[42] Melbye P, Olsson A, Hansen TH, Søndergaard HB, Bang Oturai A. Short-chain fatty acids and gut microbiota in multiple sclerosis. Acta Neurol Scand. 2019; 139:208–19. 10.1111/ane.13045 30427062

[43] Bhutia YD, Ganapathy V. Short, but Smart: SCFAs Train T Cells in the Gut to Fight Autoimmunity in the Brain. Immunity. 2015; 43:629–31. 10.1016/j.immuni.2015.09.014 26488813 PMC4930151

[44] Mizuno M, Noto D, Kaga N, Chiba A, Miyake S. The dual role of short fatty acid chains in the pathogenesis of autoimmune disease models. PLoS One. 2017; 12:e0173032. 10.1371/journal.pone.0173032 28235016 PMC5325617

[45] Rios-Covian D, González S, Nogacka AM, Arboleya S, Salazar N, Gueimonde M, de Los Reyes-Gavilán CG. An Overview on Fecal Branched Short-Chain Fatty Acids Along Human Life and as Related With Body Mass Index: Associated Dietary and Anthropometric Factors. Front Microbiol. 2020; 11:973. 10.3389/fmicb.2020.00973 32547507 PMC7271748

[46] Nagpal R, Tsuji H, Takahashi T, Nomoto K, Kawashima K, Nagata S, Yamashiro Y. Ontogenesis of the Gut Microbiota Composition in Healthy, Full-Term, Vaginally Born and Breast-Fed Infants over the First 3 Years of Life: A Quantitative Bird’s-Eye View. Front Microbiol. 2017; 8:1388. 10.3389/fmicb.2017.01388 28785253 PMC5519616

[47] Kim M, Benayoun BA. The microbiome: an emerging key player in aging and longevity. Transl Med Aging. 2020; 4:103–16. 32832742 PMC7437988

[48] de la Cuesta-Zuluaga J, Kelley ST, Chen Y, Escobar JS, Mueller NT, Ley RE, McDonald D, Huang S, Swafford AD, Knight R, Thackray VG. Age- and Sex-Dependent Patterns of Gut Microbial Diversity in Human Adults. mSystems. 2019; 4:e00261–19. 10.1128/mSystems.00261-19 31098397 PMC6517691

[49] Xu C, Zhu H, Qiu P. Aging progression of human gut microbiota. BMC Microbiol. 2019; 19:236. 10.1186/s12866-019-1616-2 31660868 PMC6819604

[50] Odamaki T, Kato K, Sugahara H, Hashikura N, Takahashi S, Xiao JZ, Abe F, Osawa R. Age-related changes in gut microbiota composition from newborn to centenarian: a cross-sectional study. BMC Microbiol. 2016; 16:90. 10.1186/s12866-016-0708-5 27220822 PMC4879732

[51] Aleman FD, Valenzano DR. Microbiome evolution during host aging. PLoS Pathog. 2019; 15:e1007727. 10.1371/journal.ppat.1007727 31344129 PMC6657895

[52] Reichardt N, Duncan SH, Young P, Belenguer A, McWilliam Leitch C, Scott KP, Flint HJ, Louis P. Phylogenetic distribution of three pathways for propionate production within the human gut microbiota. ISME J. 2014; 8:1323–35. 10.1038/ismej.2014.14 24553467 PMC4030238

[53] Weitkunat K, Schumann S, Nickel D, Kappo KA, Petzke KJ, Kipp AP, Blaut M, Klaus S. Importance of propionate for the repression of hepatic lipogenesis and improvement of insulin sensitivity in high-fat diet-induced obesity. Mol Nutr Food Res. 2016; 60:2611–21. 10.1002/mnfr.201600305 27467905 PMC5215627

[54] Bouter K, Bakker GJ, Levin E, Hartstra AV, Kootte RS, Udayappan SD, Katiraei S, Bahler L, Gilijamse PW, Tremaroli V, Stahlman M, Holleman F, van Riel NA, et al. Differential metabolic effects of oral butyrate treatment in lean versus metabolic syndrome subjects. Clin Transl Gastroenterol. 2018; 9:155. 10.1038/s41424-018-0025-4 29799027 PMC5968024

[55] Wright RS, Anderson JW, Bridges SR. Propionate inhibits hepatocyte lipid synthesis. Proc Soc Exp Biol Med. 1990; 195:26–29. 10.3181/00379727-195-43113 2399259

[56] Al-Lahham S, Roelofsen H, Rezaee F, Weening D, Hoek A, Vonk R, Venema K. Propionic acid affects immune status and metabolism in adipose tissue from overweight subjects. Eur J Clin Invest. 2012; 42:357–64. 10.1111/j.1365-2362.2011.02590.x 21913915

[57] Chambers ES, Viardot A, Psichas A, Morrison DJ, Murphy KG, Zac-Varghese SE, MacDougall K, Preston T, Tedford C, Finlayson GS, Blundell JE, Bell JD, Thomas EL, et al. Effects of targeted delivery of propionate to the human colon on appetite regulation, body weight maintenance and adiposity in overweight adults. Gut. 2015; 64:1744–54. 10.1136/gutjnl-2014-307913 25500202 PMC4680171

[58] Li Z, Yi CX, Katiraei S, Kooijman S, Zhou E, Chung CK, Gao Y, van den Heuvel JK, Meijer OC, Berbée JF, Heijink M, Giera M, Willems van Dijk K, et al. Butyrate reduces appetite and activates brown adipose tissue via the gut-brain neural circuit. Gut. 2018; 67:1269–79. 10.1136/gutjnl-2017-314050 29101261

[59] Yuille S, Reichardt N, Panda S, Dunbar H, Mulder IE. Human gut bacteria as potent class I histone deacetylase inhibitors *in vitro* through production of butyric acid and valeric acid. PLoS One. 2018; 13:e0201073. 10.1371/journal.pone.0201073 30052654 PMC6063406

[60] Jóźwiak M, Filipowska A, Fiorino F, Struga M. Anticancer activities of fatty acids and their heterocyclic derivatives. Eur J Pharmacol. 2020; 871:172937. 10.1016/j.ejphar.2020.172937 31958454

[61] Konkol Y, Keskitalo A, Vuorikoski H, Pietilä S, Elo LL, Munukka E, Bernoulli J, Tuomela J. Chronic nonbacterial prostate inflammation in a rat model is associated with changes of gut microbiota that can be modified with a galactoglucomannan-rich hemicellulose extract in the diet. BJU Int. 2019; 123:899–908. 10.1111/bju.14553 30256506

[62] European Medicines Agency. Assessment report on Serenoa repens (W. Bartram) Small, fructus. 2015.

[63] Oliphant K, Allen-Vercoe E. Macronutrient metabolism by the human gut microbiome: major fermentation by-products and their impact on host health. Microbiome. 2019; 7:91. 10.1186/s40168-019-0704-8 31196177 PMC6567490

[64] Aguirre M, Eck A, Koenen ME, Savelkoul PH, Budding AE, Venema K. Diet drives quick changes in the metabolic activity and composition of human gut microbiota in a validated *in vitro* gut model. Res Microbiol. 2016; 167:114–25. 10.1016/j.resmic.2015.09.006 26499094

[65] Szczesniak O, Hestad KA, Hanssen JF, Rudi K. Isovaleric acid in stool correlates with human depression. Nutr Neurosci. 2016; 19:279–83. 10.1179/1476830515Y.0000000007 25710209

[66] Yamashiro K, Tanaka R, Urabe T, Ueno Y, Yamashiro Y, Nomoto K, Takahashi T, Tsuji H, Asahara T, Hattori N. Gut dysbiosis is associated with metabolism and systemic inflammation in patients with ischemic stroke. PLoS One. 2017; 12:e0171521. 10.1371/journal.pone.0171521 28166278 PMC5293236

[67] Smith EA, MacFarlane GT. Enumeration of amino acid fermenting bacteria in the human large intestine: Effects of pH and starch on peptide metabolism and dissimilation of amino acids. FEMS Microbiol Ecol. 1998; 25:355–68. 10.1111/j.1574-6941.1998.tb00487.x

[68] Blachier F, Mariotti F, Huneau JF, Tomé D. Effects of amino acid-derived luminal metabolites on the colonic epithelium and physiopathological consequences. Amino Acids. 2007; 33:547–62. 10.1007/s00726-006-0477-9 17146590

[69] Jaskiewicz J, Zhao Y, Hawes JW, Shimomura Y, Crabb DW, Harris RA. Catabolism of isobutyrate by colonocytes. Arch Biochem Biophys. 1996; 327:265–70. 10.1006/abbi.1996.0120 8619613

[70] Amieva MR, Vogelmann R, Covacci A, Tompkins LS, Nelson WJ, Falkow S. Disruption of the epithelial apical-junctional complex by Helicobacter pylori CagA. Science. 2003; 300:1430–34. 10.1126/science.1081919 12775840 PMC3369828

[71] Macarthur M, Hold GL, El-Omar EM. Inflammation and Cancer II. Role of chronic inflammation and cytokine gene polymorphisms in the pathogenesis of gastrointestinal malignancy. Am J Physiol Gastrointest Liver Physiol. 2004; 286:G515–20. 10.1152/ajpgi.00475.2003 15010360

[72] González-Mariscal L, Lechuga S, Garay E. Role of tight junctions in cell proliferation and cancer. Prog Histochem Cytochem. 2007; 42:1–57. 10.1016/j.proghi.2007.01.001 17502225

[73] Feng Y, Huang Y, Wang Y, Wang P, Song H, Wang F. Antibiotics induced intestinal tight junction barrier dysfunction is associated with microbiota dysbiosis, activated NLRP3 inflammasome and autophagy. PLoS One. 2019; 14:e0218384. 10.1371/journal.pone.0218384 31211803 PMC6581431

[74] Wang HB, Wang PY, Wang X, Wan YL, Liu YC. Butyrate enhances intestinal epithelial barrier function via up-regulation of tight junction protein Claudin-1 transcription. Dig Dis Sci. 2012; 57:3126–35. 10.1007/s10620-012-2259-4 22684624

[75] Sfanos KS, Joshu CE. IBD as a risk factor for prostate cancer: what is the link? Nat Rev Urol. 2019; 16:271–72. 10.1038/s41585-019-0157-7 30742047

[76] Jaworska K, Konop M, Bielinska K, Hutsch T, Dziekiewicz M, Banaszkiewicz A, Ufnal M. Inflammatory bowel disease is associated with increased gut-to-blood penetration of short-chain fatty acids: A new, non-invasive marker of a functional intestinal lesion. Exp Physiol. 2019; 104:1226–36. 10.1113/EP087773 31243807

[77] Sun Q, Jia Q, Song L, Duan L. Alterations in fecal short-chain fatty acids in patients with irritable bowel syndrome: A systematic review and meta-analysis. Medicine (Baltimore). 2019; 98:e14513. 10.1097/MD.0000000000014513 30762787 PMC6408019

[78] Farup PG, Rudi K, Hestad K. Faecal short-chain fatty acids - a diagnostic biomarker for irritable bowel syndrome? BMC Gastroenterol. 2016; 16:51. 10.1186/s12876-016-0446-z 27121286 PMC4847229

[79] Farup PG, Valeur J. Changes in Faecal Short-Chain Fatty Acids after Weight-Loss Interventions in Subjects with Morbid Obesity. Nutrients. 2020; 12:802. 10.3390/nu12030802 32197409 PMC7146446

[80] de Bono JS, Guo C, Gurel B, De Marzo AM, Sfanos KS, Mani RS, Gil J, Drake CG, Alimonti A. Prostate carcinogenesis: inflammatory storms. Nat Rev Cancer. 2020; 20:455–69. 10.1038/s41568-020-0267-9 32546840

[81] Nickel JC. Role of prostatic inflammation in the clinical presentation of benign prostatic hyperplasia. Eur Urol Suppl. 2015; 14:e1459–63. 10.1016/S1569-9056(15)30500-5

[82] Schalken JA. Inflammation in the pathophysiology of benign prostatic hypertrophy. Eur Urol Suppl. 2015; 14:e1455–8. 10.1016/S1569-9056(15)30499-1

[83] Schenk JM, Kristal AR, Neuhouser ML, Tangen CM, White E, Lin DW, Kratz M, Thompson IM. Biomarkers of systemic inflammation and risk of incident, symptomatic benign prostatic hyperplasia: results from the prostate cancer prevention trial. Am J Epidemiol. 2010; 171:571–82. 10.1093/aje/kwp406 20142396 PMC2842217

[84] Robert G, Descazeaud A, Nicolaïew N, Terry S, Sirab N, Vacherot F, Maillé P, Allory Y, de la Taille A. Inflammation in benign prostatic hyperplasia: a 282 patients’ immunohistochemical analysis. Prostate. 2009; 69:1774–80. 10.1002/pros.21027 19670242 PMC2833181

[85] Krušlin B, Tomas D, Džombeta T, Milković-Periša M, Ulamec M. Inflammation in Prostatic Hyperplasia and Carcinoma-Basic Scientific Approach. Front Oncol. 2017; 7:77. 10.3389/fonc.2017.00077 28487844 PMC5403898

[86] Pace G, Di Massimo C, De Amicis D, Vicentini C, Ciancarelli MG. Inflammation and endothelial activation in benign prostatic hyperplasia and prostate cancer. Int Braz J Urol. 2011; 37:617–22. 10.1590/s1677-55382011000500008 22099274

[87] Bardan R, Dumache R, Dema A, Cumpanas A, Bucuras V. The role of prostatic inflammation biomarkers in the diagnosis of prostate diseases. Clin Biochem. 2014; 47:909–15. 10.1016/j.clinbiochem.2014.02.008 24560954

[88] Cai T, Santi R, Tamanini I, Galli IC, Perletti G, Bjerklund Johansen TE, Nesi G. Current Knowledge of the Potential Links between Inflammation and Prostate Cancer. Int J Mol Sci. 2019; 20:3833. 10.3390/ijms20153833 31390729 PMC6696519

[89] Verdugo-Meza A, Ye J, Dadlani H, Ghosh S, Gibson DL. Connecting the Dots Between Inflammatory Bowel Disease and Metabolic Syndrome: A Focus on Gut-Derived Metabolites. Nutrients. 2020; 12:1434. 10.3390/nu12051434 32429195 PMC7285036

[90] Park S, Kang J, Choi S, Park H, Hwang E, Kang YG, Kim AR, Holzapfel W, Ji Y. Cholesterol-lowering effect of Lactobacillus rhamnosus BFE5264 and its influence on the gut microbiome and propionate level in a murine model. PLoS One. 2018; 13:e0203150. 10.1371/journal.pone.0203150 30153290 PMC6112659

[91] Tirosh A, Calay ES, Tuncman G, Claiborn KC, Inouye KE, Eguchi K, Alcala M, Rathaus M, Hollander KS, Ron I, Livne R, Heianza Y, Qi L, et al. The short-chain fatty acid propionate increases glucagon and FABP4 production, impairing insulin action in mice and humans. Sci Transl Med. 2019; 11:eaav0120. 10.1126/scitranslmed.aav0120 31019023

[92] Heimann E, Nyman M, Pålbrink AK, Lindkvist-Petersson K, Degerman E. Branched short-chain fatty acids modulate glucose and lipid metabolism in primary adipocytes. Adipocyte. 2016; 5:359–68. 10.1080/21623945.2016.1252011 27994949 PMC5160390

[93] Granado-Serrano AB, Martín-Garí M, Sánchez V, Riart Solans M, Berdún R, Ludwig IA, Rubió L, Vilaprinyó E, Portero-Otín M, Serrano JC. Faecal bacterial and short-chain fatty acids signature in hypercholesterolemia. Sci Rep. 2019; 9:1772. 10.1038/s41598-019-38874-3 30742005 PMC6370822

[94] Fu J, Bonder MJ, Cenit MC, Tigchelaar EF, Maatman A, Dekens JA, Brandsma E, Marczynska J, Imhann F, Weersma RK, Franke L, Poon TW, Xavier RJ, et al. The Gut Microbiome Contributes to a Substantial Proportion of the Variation in Blood Lipids. Circ Res. 2015; 117:817–24. 10.1161/CIRCRESAHA.115.306807 26358192 PMC4596485

[95] Roelofsen H, Priebe MG, Vonk RJ. The interaction of short-chain fatty acids with adipose tissue: relevance for prevention of type 2 diabetes. Benef Microbes. 2010; 1:433–37. 10.3920/BM2010.0028 21831781

[96] Fernandes J, Su W, Rahat-Rozenbloom S, Wolever TM, Comelli EM. Adiposity, gut microbiota and faecal short chain fatty acids are linked in adult humans. Nutr Diabetes. 2014; 4:e121. 10.1038/nutd.2014.23 24979150 PMC4079931

[97] Hald S, Schioldan AG, Moore ME, Dige A, Lærke HN, Agnholt J, Bach Knudsen KE, Hermansen K, Marco ML, Gregersen S, Dahlerup JF. Effects of Arabinoxylan and Resistant Starch on Intestinal Microbiota and Short-Chain Fatty Acids in Subjects with Metabolic Syndrome: A Randomised Crossover Study. PLoS One. 2016; 11:e0159223. 10.1371/journal.pone.0159223 27434092 PMC4951149

[98] Upadhyaya B, McCormack L, Fardin-Kia AR, Juenemann R, Nichenametla S, Clapper J, Specker B, Dey M. Impact of dietary resistant starch type 4 on human gut microbiota and immunometabolic functions. Sci Rep. 2016; 6:28797. 10.1038/srep28797 27356770 PMC4928084

[99] Cani PD. Microbiota and metabolites in metabolic diseases. Nat Rev Endocrinol. 2019; 15:69–70. 10.1038/s41574-018-0143-9 30602737

[100] Alberti KG, Zimmet P, Shaw J. Metabolic syndrome--a new world-wide definition. A Consensus Statement from the International Diabetes Federation. Diabet Med. 2006; 23:469–80. 10.1111/j.1464-5491.2006.01858.x 16681555

