# Supplementary material for: Alterations in fecal short chain fatty acids (SCFAs) and branched short-chain fatty acids (BCFAs) in men with benign prostatic hyperplasia (BPH) and metabolic syndrome (MetS)
Source: Aging (Albany NY). 2021 Apr 13;13(8):10934–54. doi: 10.18632/aging.202968 (PMC8109139; doi:10.18632/aging.202968)
Supplement: Supplementary Tables [file aging-13-202968-s001.pdf]

## SUPPLEMENTARY TABLES

**Supplementary Table 1. Correlation between short-chain fatty acids and BMI, depending on the diagnosis of BPH.**

| SCFAs (%) | Healthy volunteers (without BPH) (n=80) | | Patients with BPH (n=103) | |
|---|---|---|---|---|
| | BMI | | | |
| | R | p | R | p |
| **C 2:0** | **-0.221** | **0.048*** | 0.061 | 0.546 |
| **C 3:0** | 0.010 | 0.931 | -0.062 | 0.542 |
| **C 4:0 i** | -0.015 | 0.894 | -0.144 | 0.152 |
| **C 4:0 n** | 0.057 | 0.617 | 0.094 | 0.352 |
| **C 5:0 i** | -0.006 | 0.960 | -0.180 | 0.072 |
| **C 5:0 n** | **0.287** | **0.010*** | -0.129 | 0.201 |
| **C 6:0 i** | 0.000 | 0.997 | **-0.246** | **0.014*** |
| **C 6:0 n** | 0.104 | 0.359 | -0.040 | 0.690 |

BPH – benign prostatic hyperplasia; BMI – body mass index; SCFAs – short chain fatty acids; C2:0 - acetic acid; C3:0 - propionic acid; C4:0i - isobutyric acid; C4:0n - butyric acid; C5:0i - isovaleric acid; C5:0n - valeric acid; C6:0i – isocaproic acid; C 6:0n - caproic acid; R - correlation coefficient; p – statistical significance; ***** - statistical significant parameter.

**Supplementary Table 2. Correlation between short chain fatty acids and BMI, depending on the occurrence of metabolic syndrome in all patients participating in the study (patients without BPH and with BPH).**

| SCFAs (%) | All participants | | | |
|---|---|---|---|---|
| | Without MetS (n=105) | | With MetS (n=78) | |
| | BMI | | | |
| | R | p | R | p |
| **C 2:0** | -0.082 | 0.412 | 0.019 | 0.869 |
| **C 3:0** | -0.014 | 0.890 | **-0.265** | **0.020*** |
| **C 4:0 i** | -0.126 | 0.203 | 0.040 | 0.730 |
| **C 4:0 n** | 0.155 | 0.118 | 0.044 | 0.702 |
| **C 5:0 i** | -0.131 | 0.188 | 0.018 | 0.875 |
| **C 5:0 n** | 0.039 | 0.693 | 0.057 | 0.622 |
| **C 6:0 i** | -0.053 | 0.592 | -0.081 | 0.483 |
| **C 6:0 n** | -0.017 | 0.861 | 0.206 | 0.072 |

MetS – metabolic syndrome; SCFAs – short chain fatty acids; C2:0 - acetic acid; C3:0 - propionic acid; C4:0i - isobutyric acid; C4:0n - butyric acid; C5:0i - isovaleric acid; C5:0n - valeric acid; C6:0i - isocaproic acid; C 6:0n - caproic acid; R - correlation coefficient; p – statistical significance; ***** - statistical significant parameter.

**Supplementary Table 3. Correlation between short-chain fatty acids and BMI and the occurrence of metabolic syndrome in healthy volunteers and BPH patients.**

| SCFAs (%) | Healthy volunteers (without BPH) n=80 | | | | Patients with BPH (n=103) | | | |
|---|---|---|---|---|---|---|---|---|
| | Without MetS (n=44) | | With MetS (n=36) | | Without MetS (n=61) | | With MetS (n=42) | |
| | BMI | | | | BMI | | | |
| | R | p | R | p | R | p | R | p |
| **C 2:0** | -0.182 | 0.238 | -0.199 | 0.245 | -0.049 | 0.710 | 0.208 | 0.192 |
| **C 3:0** | 0.005 | 0.975 | **-0.517** | **0.001*** | -0.069 | 0.600 | -0.128 | 0.424 |
| **C 4:0 i** | 0.026 | 0.864 | 0.193 | 0.260 | -0.190 | 0.147 | 0.001 | 0.995 |
| **C 4:0 n** | 0.063 | 0.684 | 0.267 | 0.115 | 0.217 | 0.096 | -0.074 | 0.646 |
| **C 5:0 i** | 0.038 | 0.807 | 0.158 | 0.356 | -0.225 | 0.084 | -0.022 | 0.890 |
| **C 5:0 n** | 0.205 | 0.183 | 0.168 | 0.329 | -0.064 | 0.628 | -0.099 | 0.539 |
| **C 6:0 i** | 0.071 | 0.645 | 0.046 | 0.791 | -0.111 | 0.398 | -0.220 | 0.167 |
| **C 6:0 n** | 0.043 | 0.782 | **0.329** | **0.050*** | -0.076 | 0.563 | 0.097 | 0.546 |

BPH – benign prostatic hyperplasia; MetS – metabolic syndrome; BMI – body mass index; SCFAs – short chain fatty acids; C2:0 - acetic acid; C3:0 - propionic acid; C4:0i - isobutyric acid; C4:0n - butyric acid; C5:0i - isovaleric acid; C5:0n - valeric acid; C6:0i – isocaproic acid; C 6:0n - caproic acid; R - correlation coefficient; p* - statistical significant parameter.
